# Supplementary material for: Beige adipocytes mediate the neuroprotective and anti-inflammatory effects of subcutaneous fat in obese mice
Source: Nat Commun. 2021 Jul 30;12:4623. doi: 10.1038/s41467-021-24540-8 (PMC8324783; doi:10.1038/s41467-021-24540-8)
Supplement: Supplementary file 1 — Supplementary Information [file 41467_2021_24540_MOESM1_ESM.pdf]

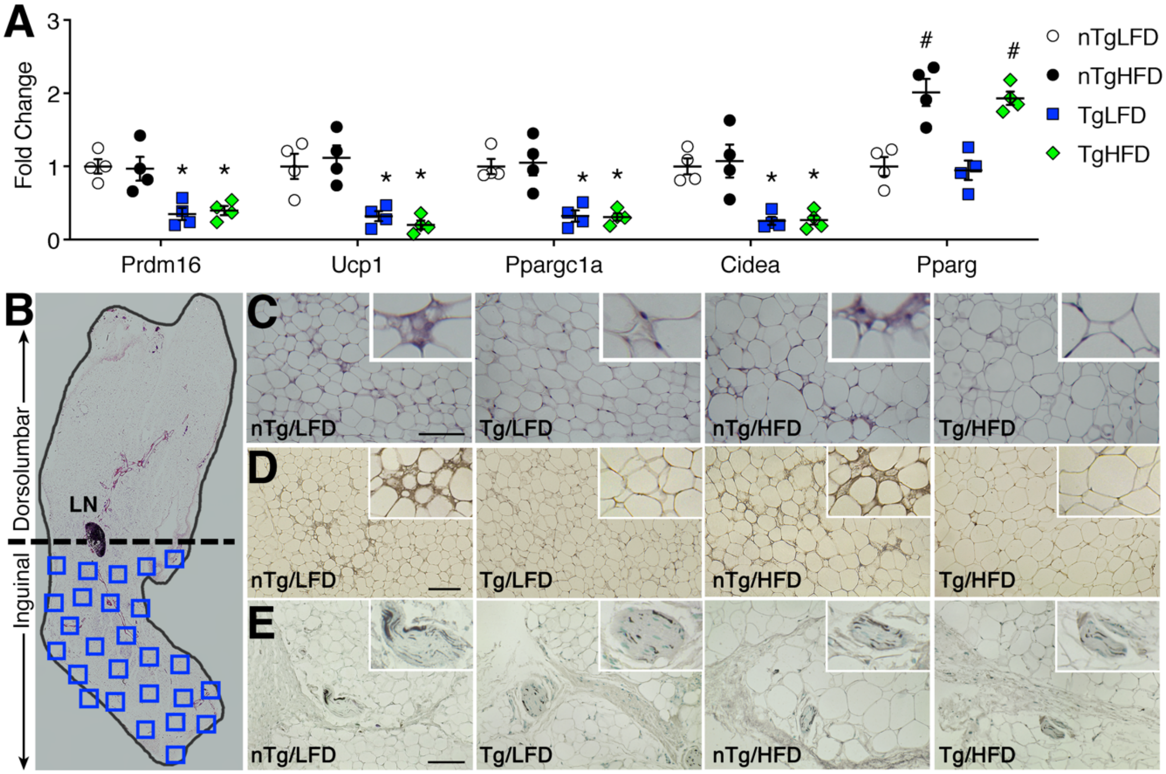

Supplemental Figure 1. Loss of thermogenic signatures in SAT from mice lacking beige fat. A) Reductions in beiging-associated gene expression in inguinal SAT from Adiponectin^{cre}/PRDM16$^{fl/fl}$ transgenic (Tg) mice and nontransgenic (nTg) littermates maintained on low-fat or high-fat diet (LFD, HFD) for 4wk. Symbols show data from individual mice and lines show mean±sem (n=4/condition). B) Systematic random sampling of inguinal SAT for analysis of multilocular adipocytes, UCP1+ adipocytes, and tyrosine hydroxylase (TH)-labeled fibers. Images were sampled from (n=4) mice/condition (applies to panels B-E). C) Representative micrographs from HNE-stained sections in the indicated conditions. D) Immunostaining for UCP1 in the indicated groups. E) Immunostaining for TH in the indicated conditions. For panels (C-E), scalebar shown for nTg/LFD = 100 microns and applies to all micrographs. For A), data were analyzed with 2-way ANOVA; *effect of genotype ($p<0.05$); $^{\#}$effect of diet by Tukey's multiple comparison test . For For data, statistics, and exact p-values, see Source Data File 7.

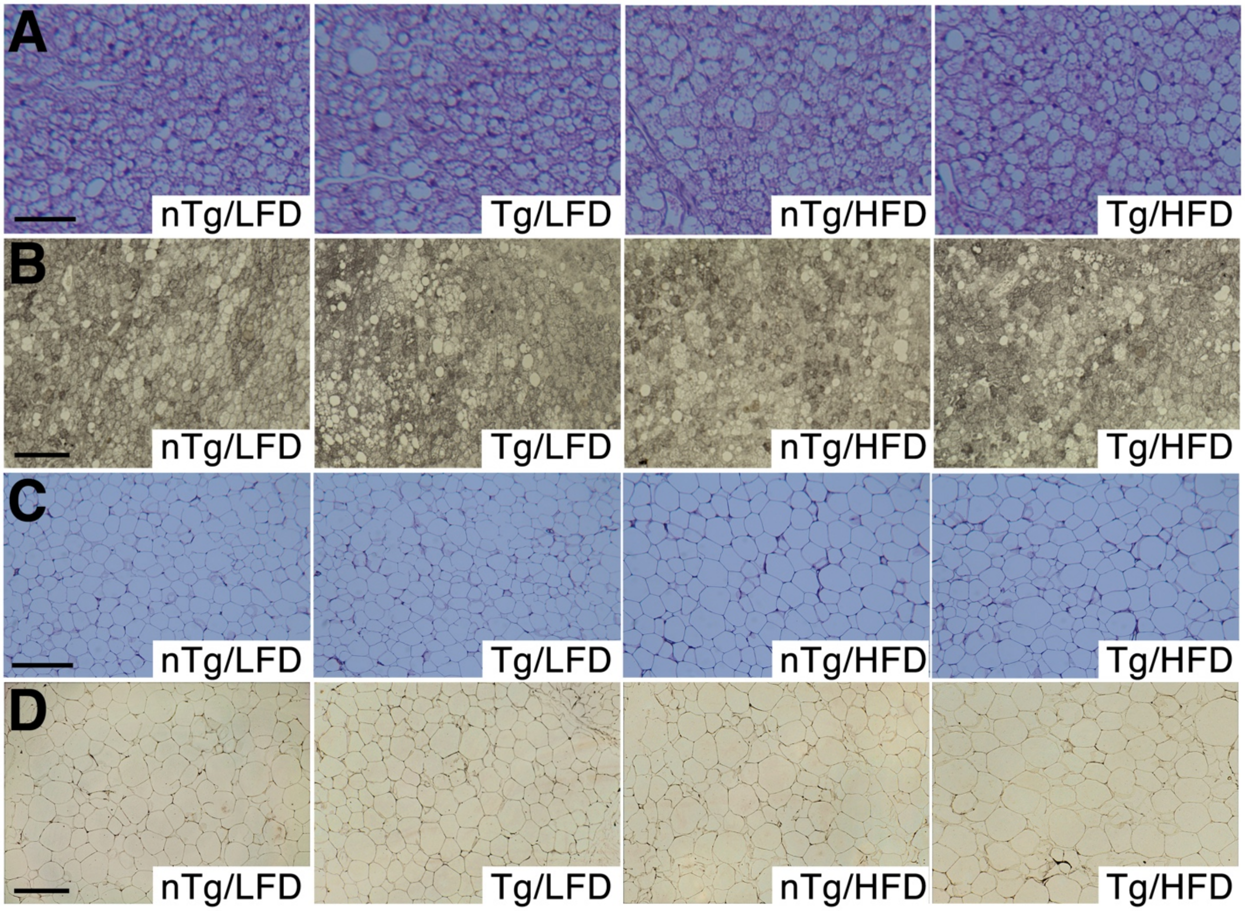

Supplemental Figure 2. No changes in morphology or UCP1 immunoreactivity in BAT or VAT from mice lacking beige fat. A) Representative micrographs of HNE staining from interscapular BAT in the indicated conditions. B) Immunostaining for UCP1 in interscapular BAT. C) Micrographs of HNE staining in epididymal VAT from the indicated conditions. D) UCP1 immunostaining in VAT. For all panels, scalebar shown for nTg/LFD = 100 microns and applies to all images of that stain or antigen. Samples were stained and imaged from (n=4) mice/condition.

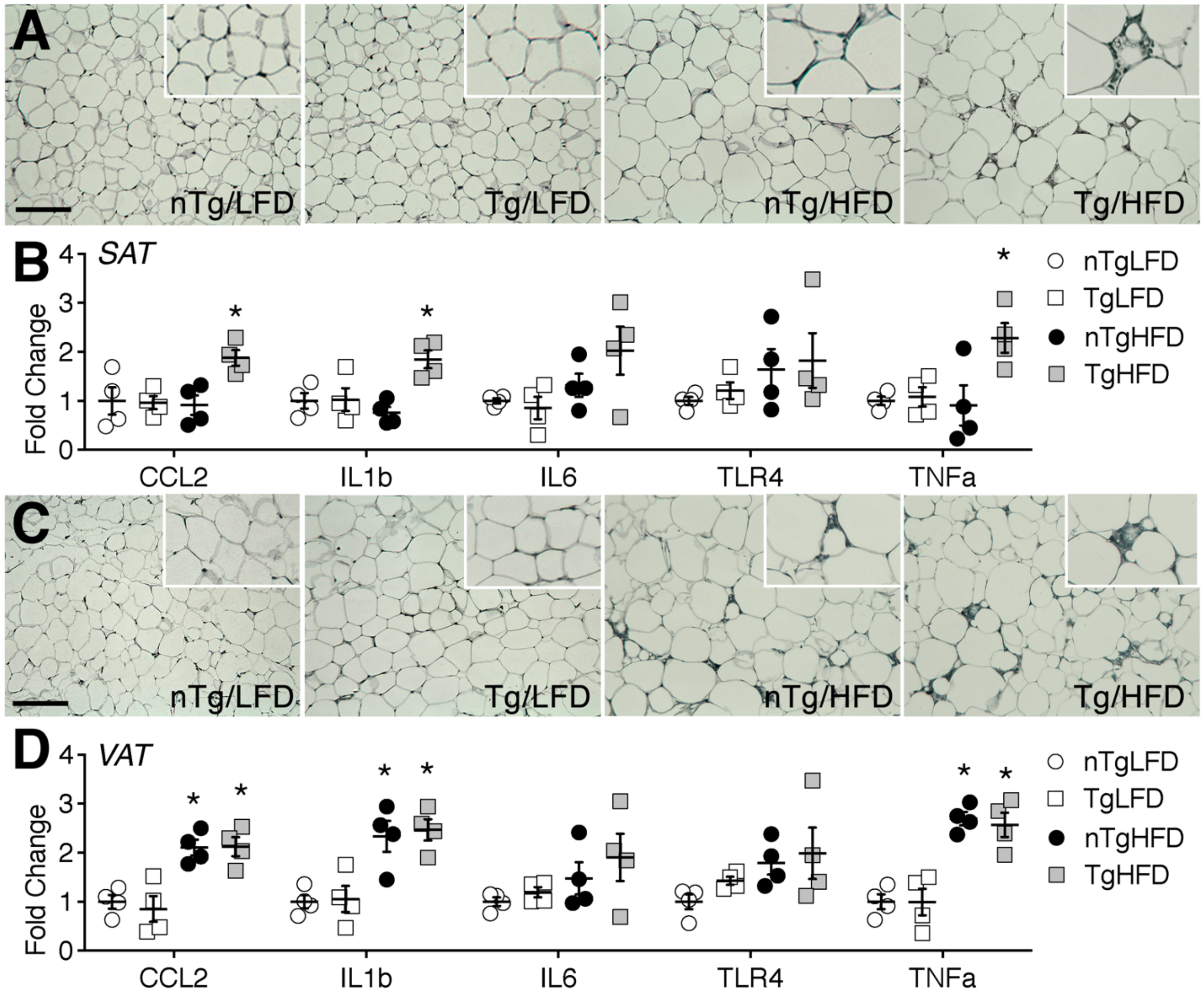

Supplementary Figure 3. Acquisition of VAT-like properties in SAT from beige fat knockout mice with dietary obesity. A) Representative micrographs of F4/80+ crown-like structures in SAT from the indicated conditions. B) Pro-inflammatory cytokine gene expression in SAT. C) Representative micrographs of F4/80+ crown-like structures in VAT from each condition. D) Pro-inflammatory cytokine gene expression in VAT. For graphs (B, D), symbol height represents data from individual mice, lines depict group mean (n=4 per condition), and error bars represent sem. Data were analyzed with 2-way ANOVA; *significant difference relative to nTgLFD ($p<0.05$) by Tukey's multiple comparison test. For panels (A, C), scalebar shown for nTg/LFD = 100 microns and applies to all micrographs. Samples were stained and imaged from (n=4) mice/condition. For image acquisition and sampline parameters, see Supplementary Methods. For data, statistics, and exact p-values, see Source Data File 7.

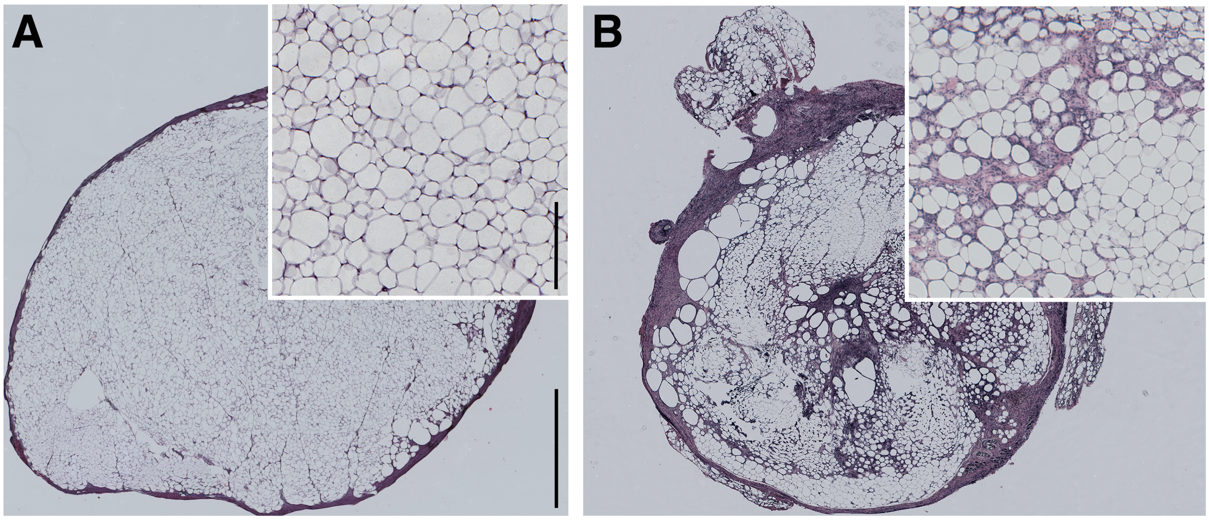

Supplemental Figure 4. Histological assessment of transplant viability. Micrographs of HNE-stained sections were acquired as brightfield montages. Healthy transplants (A) were identified by the absence of fibrotic and necrotic tissue throughout the transplant. Rejected transplants (B) were identified based on widespread immune cell infiltration, interstitial fibrosis, and irregular adipocyte diameters. Scalebar (A) = 2.0mm; inset scalebar = 100 microns. Transplants from all (n=107) recipient mice in this report were processed for paraffin embedding and checked for viability (see Supplemental Methods for criteria). Nine mice were excluded due to transplant rejection.

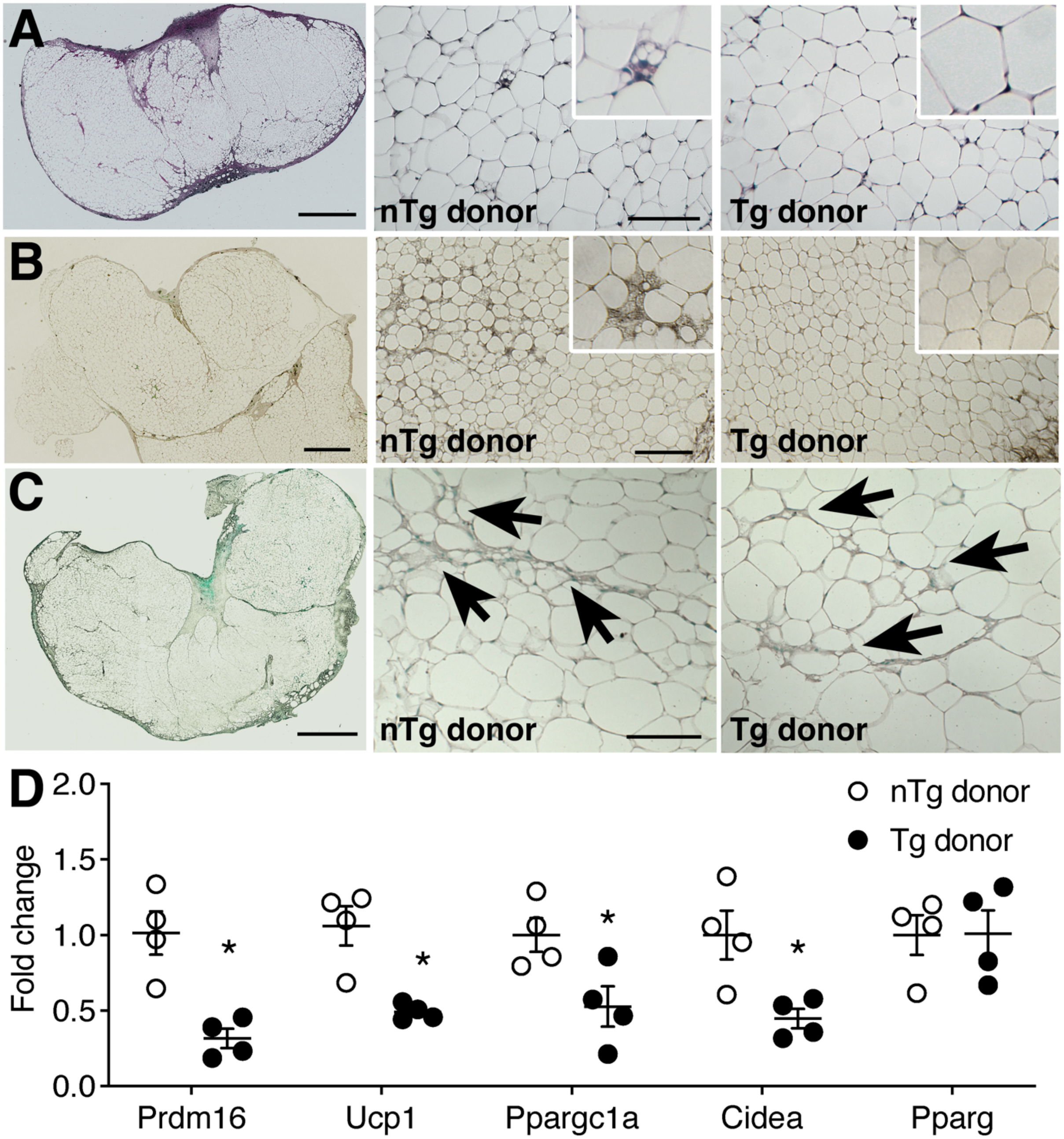

Supplementary Figure 5. Persistence of beige adipocytes and thermogenic gene expression in transplanted SAT. A) Multilocular adipocytes on HNE-stained sections of transplanted SAT from a nontransgenic (nTg; n=4) or Adiponectin^{cre}/PRDM16$^{fl/fl}$ transgenic (Tg; n=4) donor. Transplants were collected at sacrifice from C57Bl6J (Wt) recipient mice with high-fat diet-induced obesity and imaged as reported in Supplementary Methods. Scalebar shown for montage images (far left) = 2.0mm.

Scalebar shown for nTg donor micrographs (center) = 100 microns and Tg donor micrographs were captured at the same scaling. Scalebar lengths apply to (A-C). B) Representative micrographs of UCP1 immunostaining in transplanted SAT from nTg or Tg donors. C) Micrographs of immunostaining for tyrosine hydroxylase (TH) in transplanted SAT from nTg or Tg donor. D) Expression of beiging-associated genes in transplanted SAT from nTg or Tg donors. Symbols represent data from individual mice and lines depict mean±sem (n=4/condition). For D), asterisk indicates the effect of donor genotype at $p<0.05$ determined using bidirectional t-tests. For data, statistics, and exact p-values, see Source Data File 7.

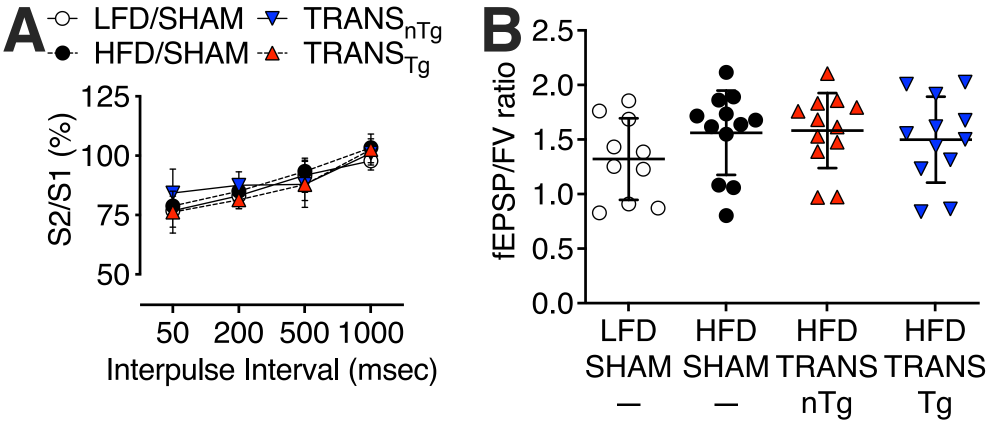

Supplemental Figure 6. Stability of presynaptic plasticity and input/output ratios after SAT transplantation in obese mice. A) Paired-pulse depression, indicated by reductions in the slope of a second field excitatory postsynaptic potential (S2) at intervals following the first (S1), is unaffected by diet or SAT transplantation. Symbols and error bars show group mean±sem. B) No effect of diet or surgery on relationships between fEPSP slope and fiber volley (FV) amplitude across a range of stimulus intensities. Symbol height represents data from individual slice recordings and lines shows group mean±sem. For LFD/SHAM, n=10 slice recordings from 5 mice; for all other groups, n=12 slice recordings from 6 mice (applies to A-B). See Source Data File 7.

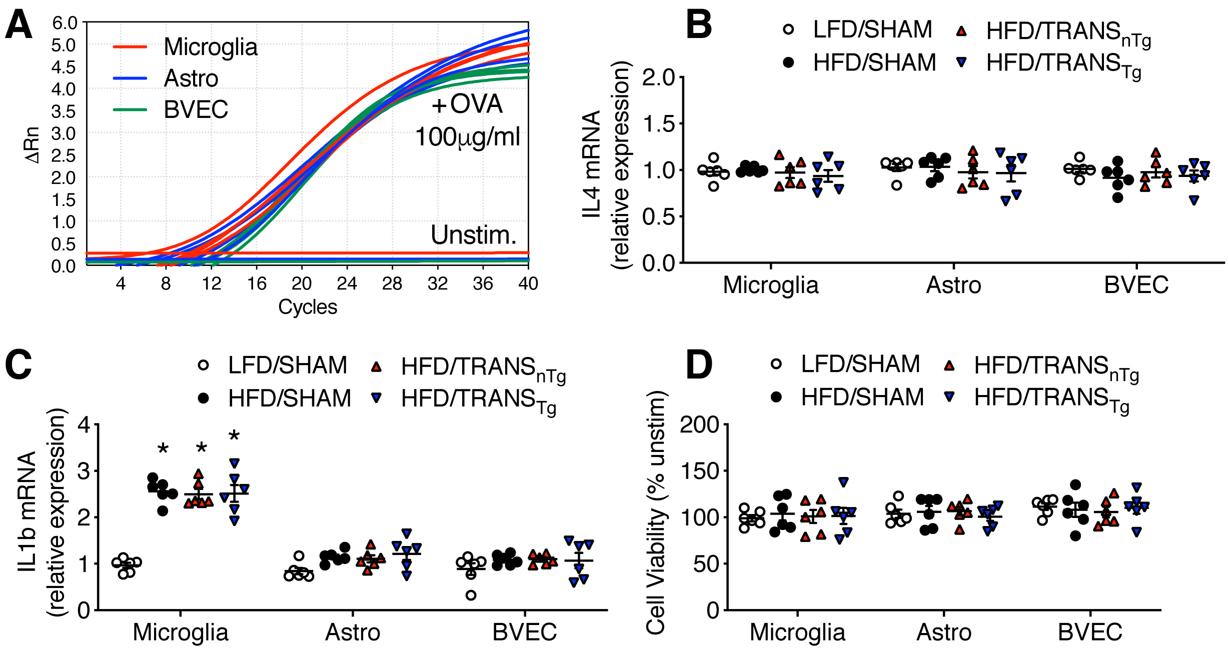

Supplemental Figure 7. SAT transplantation does not promote basal synthesis of IL4 among cells of the brain parenchyma. A) IL4 mRNA is not detectable in primary microglia (Mic), astrocytes (Astro), or brain vascular endothelial cells (BVEC) under unstimulated conditions (Unstim), but incubation with ovalbumin (OVA) induced IL4 expression in all cell types. B) OVA-stimulated IL4 expression is unaffected by obesity, SAT transplantation, or donor genotype. C) Obesity enhances OVA-stimulated IL1b gene expression in primary microglia, irrespective of SAT transplantation or donor genotype. D) No differences in cell viability after OVA stimulation. For all graphs, symbols represent data from individual mice, line shows the average of (n=6) per condition, error bars indicate sem, and asterisk denotes statistically significant effect of diet at p<0.05 by ANOVA w/Tukey's post hoc. For data, statistics, and exact p-values, see Source Data File 7.

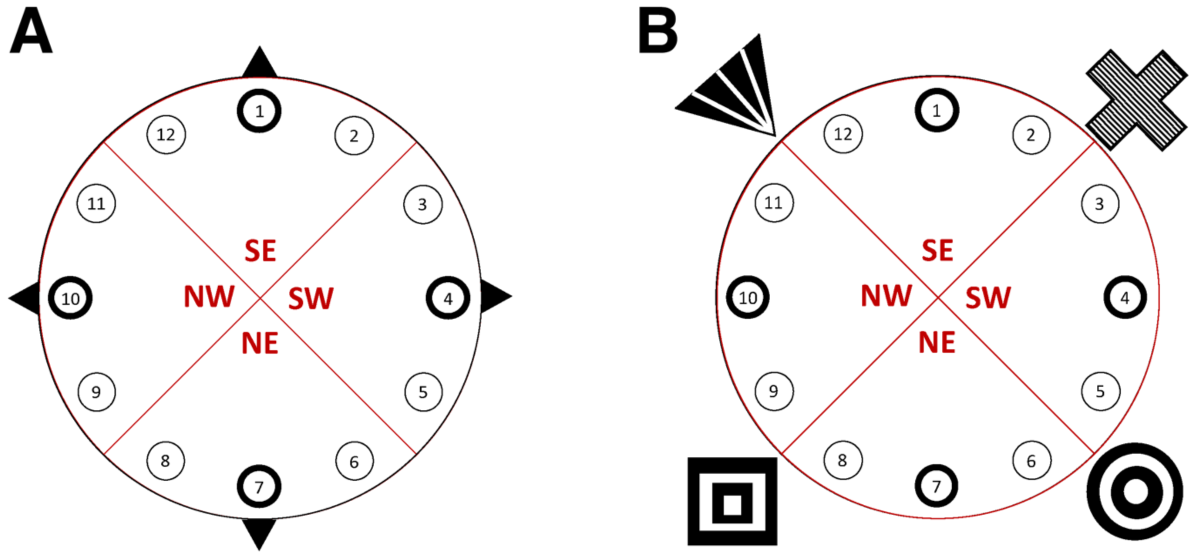

Supplementary Figure 8. Apparatus layout for Barnes maze testing. A) During the first two days of training, the escape hole was marked by a proximal cue (indicated by the black triangle) attached to the circular platform above the location of the escape box. Each mouse received (4) trials per day with the escape box located under hole #1, in the southeast quadrant (SE); hole #4, in the southwest quadrant (SW); hole #7, in the northeast quadrant (NE); and hole #10, in the northwest quadrant (NW). The sequence of escape box locations during training with the proximal cue was randomized such that each experimental condition contained equal numbers of mice trained according to the following sequence: SE/SW/NE/NW, NW/SE/SW/NE; NE/NW/SE/SW; SW/NE/NW/SE (n=12 mice per condition). B) In preparation for days 3-6 of training, equal numbers of mice from each condition were randomly assigned to be trained with the escape box under hole #1 (SE), #4 (SW), #7 (NE), or #10 (NW). The assigned escape box location was the same throughout days 3-6.

Supplementary Methods

Behavioral tests

Novel object preference testing began with 10min video analysis of locomotor behavior in the arena without objects, followed by 10min video tracking of interaction with two identical objects. After a 30min delay, mice were reintroduced to the arena with one novel and one familiar object for 5min video tracking. This sequence was repeated 2hr and 24hr post-training, with the position of the novel and familiar objects varied across trials. For testing in the Y-maze, mice were confined to the center of the maze for 5min, followed by video tracking of spontaneous alternation until completion of seven arm entries. Mice that jumped out of the Y-maze apparatus before completing seven arm entries were returned to the home cage and retested 1.5-2.0hr later. For the water maze, mice initially received four 90sec trials with a visible platform and no intra-maze cues. For hidden platform training, intramaze cues were attached to the walls of the pool and mice received four trials per day over five consecutive days (15min inter-trial interval). After hidden platform training, mice received a single 90sec probe trial without the platform.

For the Barnes maze, mice were tested on a white circular platform (122cm diameter) with twelve holes (9cm diameter) evenly spaced around the perimeter (each hole 5cm from edge). The circular platform was surrounded by a curtain to prevent the use of distal cues. Each trial began with 30sec under the start box in the center of the maze, followed by 2min free exploration. Mice that did not locate the escape box within 2min were guided there by the experimenter and all mice remained in the escape box for 1min after each trial. The apparatus was cleaned with fragrance-free Bac-Down disinfectant (Decon Labs, King of Prussia, PA USA) between trials. Testing was carried

out during the dark phase under dim white light illumination, and an auditory stimulus (white noise, 60dB x 1sec) was delivered when mice stopped moving for >3sec.

During the first two training days, the escape box was marked with a proximal visual cue in one of four locations (Supplementary Fig.8A). Each mouse received (4) trials per day with the escape box located under hole #1, in the southeast quadrant (SE); hole #4, in the southwest quadrant (SW); hole #7, in the northeast quadrant (NE); and hole #10, in the northwest quadrant (NW). The sequence of escape box locations during training with the proximal cue was randomized such that each experimental condition contained equal numbers of mice trained according to the following sequence: SE/SW/NE/NW, NW/SE/SW/NE; NE/NW/SE/SW; SW/NE/NW/SE.

During days 3-6, mice were trained to locate the escape box based on the location of extramaze cues (Supplementary Fig.8B). In preparation for days 3-6 of training, equal numbers of mice from each condition were randomly assigned to training with the escape box under hole #1 (SE), #4 (SW), #7 (NE), or #10 (NW). The assigned escape box location was the same throughout days 3-6. The extramaze cues were high-contrast concentric black-and-white squares, circles, triangles, or crosses measuring 20cm high and 18-20cm wide. Extramaze cues were suspended around the perimeter of the maze 6-8cm above the surface of the platform (without touching the platform). Cues were not suspended directly above the location of the escape box. On day 7, mice received a 2min probe trial without the escape box. For data analysis and visualization purposes (e.g. Fig.2F), target location during the probe trial was aligned over hole #6 for all animals. For all behavioral tests, animals were tracked using Any-maze software (Stoelting).

Intraperitoneal glucose tolerance testing

Intraperitoneal glucose tolerance testing was carried out after an overnight fast, as reported previously.[1] To summarize, baseline samples were collected from the tail vein using a handheld glucometer immediately before intraperitoneal administration of glucose in sterile saline (1.0g/kg; Sigma-Aldrich). Subsequent samples were collected 30min, 60min, and 120min after glucose administration. Tail-nicks were cleaned with Betadine and sealed with styptic powder before returning the mouse to its home cage.

Electrophysiology

Acute slices (350 micron thickness) were prepared on a Vibratome and allowed to recover for at least 1hr in carboxygenated artificial cerebrospinal fluid (ACSF) before recording, as reported previously.[2-3] Medial perforant path projections to the dentate gyrus were identified based on their location in the middle molecular layer and on the presence of paired-pulse depression, which was also quantified across a range of interstimulus intervals as a measure of presynaptic plasticity. Baseline stimulation at 50% of maximal response was carried out for at least 10min at 0.05Hz. LTP was induced with a 1sec train delivered at 100Hz and post-induction responses were recorded for at least 60min at 0.05Hz. For some experiments, slices were preincubated with minocycline (20 micromolar) or neutralizing antibody against IL4 (IL-4 nAb, 5 micrograms/mL; rat anti-mouse IL-4, clone 11B11; Invitrogen, Carlsbad, CA) for 30min with continuous application throughout recording. LTP was quantified as percent change in fEPSP slope during minutes 50-60 of recording relative to pre-tetanus baseline and a paired t-test was applied to determine whether potentiation had occurred. Data from slices that did not potentiate

was discarded, as was data from slices that exhibited >10% variability during baseline recording.

Adipose transplantation surgery and transplant viability

For subcutaneous fat transplantation, recipients and donors (6-8wk old lean males) were maintained under Isoflurane anesthetic in side-by-side aseptic surgical stations. Each inguinal fat pad was unilaterally excised from the donor, starting from the dorsolumbar region to just above the femoral artery. The excised fat was trimmed to 250mg and secured against the inner surface of the peritoneal wall with a dissolving suture, as reported previously for VAT transplantation.[2-3] Parallel groups of mice received sham operation involving all procedures except transplant insertion. At euthanasia, transplants were collected and fixed in 4% paraformaldehyde (PFA) for 24hr before paraffin embedding and sectioning.

Immunohistochemistry in paraffin sections from adipose tissue

Immunoperoxidase histochemistry was performed on paraffin sections from adipose tissue. Embedding and sectioning (9 micron thickness) was carried out with assistance from the Augusta University Histology Core. Slides were deparaffinized through Xylenes, rehydrated through decreasing concentrations of ethanol, and washed overnight in 0.1M PBS. The next day, endogenous peroxidases were quenched in 0.3% H2O2 in PBS (v/v), washed 3x in PBS, and unmasked in 0.1M citric acid (pH=6.0). After antigen unmasking, slides were washed in PBS before 24-48hr incubation in primary antibodies against UCP1 (Abcam catalog #ab10983; 1:1,000), tyrosine hydroxylase

(Millipore catalog #AB152, 1:1,000), or F4/80 (Santa Cruz catalog #sc25830, 1:500). After primary antibody incubation, slides were washed 3x in PBS before incubation with biotinylated secondary antibodies (Vector Elite catalog #6101, 1:200) diluted in Superblock (Thermo Fisher Pierce catalog #37515) for 1hr. Following additional washes, biotinylated secondary antibodies were amplified with avidin-biotin complex (Vector Elite catalog #6101, 1:100) for 1hr before additional washes and visualization with diaminobenzadine (Vector catalog #SK-4100). For TH and F4/80, slides were counterstained with Methyl Green before dehydration and coverslipping under Permount.

Adipose tissue image analysis

Quantitative stereological analysis of paraffin-embedded samples requires thick sections and randomization of tissue orientation prior to sectioning.[4-5] Because the current series of studies used thin sections and did not randomize tissue orientation, we used a semiquantitative physical fractionator approach for analysis. Tissue sections were traced in StereoInvestigator software (Microbrightfield, Williston VT) using a Zeiss upright microscope equipped with a motorized stage. The systematic random sampling scheme was set to acquire 2-dimensional fields covering 50% of the traced area at 20X magnification with manual plane-of-focus calibration. This approach generated a series of (500-700) images from (2-4) sections per animal.

Image series were loaded into ImageJ as stacks for blinded visual inspection and analysis to determine the presence or absence of features of interest (HNE, multilocular adipocytes; UCP1, immunostained multilocular adipocytes; TH, stained fibers or fiber bundles; F4/80, crown-like structures). For each of the preceding stains or markers, the

number of fields with relevant features of interest was expressed as percent of total sampled fields for comparison.

RNA extraction from paraffin-embedded tissue

Extraction of RNA from paraffin-embedded adipose tissues was carried out using the RNeasy FFPE kit (Qiagen), as described,[2] with one exception. To account for anatomical variability in the capacity for 'beiging' in dorsolumbar versus inguinal SAT,[6] the inguinal lymph node was identified on each paraffin block with the aid of a dissecting microscope. If the lymph node was not visible, blocks were trimmed until it became visible. Tissue orientation was confirmed using micrographs of HNE staining from adjacent sections and (4) 2.0mm tissue punches were collected from inguinal SAT below the lymph node. cDNA was synthesized from RNA template using a commercially available kit according to the manufacturer's instructions (Applied Biosystems). cDNA samples (500ng) were amplified using Taqman probes, with the average dCT for nTg/LFD (Supplementary Fig.1, Supplementary Fig.3) or nTg donor samples (Supplementary Fig.5) as the reference group for calculating relative expression. For a list of Taqman probes used in these experiments, see Supplementary Table 1.

In vivo capture and quantification of interleukin-4

Mice received bilateral injections of azide-free, low endotoxin biotinylated rat anti-mouse IL-4 into the lateral ventricles (1.0 microgram; clone BVD4-1D11; BD Bioscience) at the following coordinates: AP -1.6; ML ±2.3; DV+2.2 (atlas of Paxinos and Franklin[7]) using a Kopf stereotaxic frame equipped with a Neuros syringe (210 micron tip diameter;

Hamilton Company, Reno, NV USA). For IP administration, non-overlapping groups of mice were injected with 10 micrograms of the capture antibody in 0.2mL sterile saline. One day after administration of the capture antibody, mice were anesthetized for CSF collection via the cisterna magna or decapitated for blood collection and serum separation. Samples were snap-frozen and stored at -80 in preparation for ELISA. Plates were coated with unlabeled rat anti-mouse IL-4 (clone BVD6-24G2.3; Southern Biotech, Birmingham, AL USA), and recombinant mouse IL-4 (Shenandoah Biotechnologies) was used to generate the standard curve, as described.[8]

Isolation of stromal-vascular fraction from adipose tissue

For collagenase digestion and isolation of stromal-vascular fraction (SVF) from adipose tissue (Fig.6c), transplanted SAT, resident SAT, and VAT were dissected, weighed, and washed in DMEM to remove hair and debris. Visible blood vessels were removed and discarded, then fat pads were minced and transferred to conical tubes. Dissociation buffer (1xdPBS with 2.0mg/mL collagenase IV and 20mg/mL BSA; 3x wt/vol) was added to each tube and samples dissociated for 30-40min at 37C on a shaker inside of a hybridization oven. After dissociation, 3x (vol/vol) DMEM+2%FBS was added to each tube. Cell suspensions were passed through a 100 micron strainer and pelleted by centrifugation at 250xg for 8min. The lipid layer and media were aspirated and discarded, and the SVF pellet was resuspended in 5mL DMEM+FBS. The SVF pellet was filtered through a 40 micron strainer, pelleted by centrifugation at 400xg for 10min, then washed 2x by repeated resuspension and centrifugation before antibody labeling and flow cytometry.

Forebrain cell isolations

For isolation of forebrain mononuclear cells (FMCs), brains were extracted from the skull after transcardial perfusion with saline, as described.[2-3] After removing the cerebellum and brainstem, forebrain hemispheres were bisected and midline white matter tracts were removed. Each hemisphere was manually dissociated in an ice-cold Tenbroeck homogenizer containing 5.0mL sterile Dulbecco's phosphate-buffered saline (dPBS) supplemented with 0.2% D-glucose. Dissociated cells were passed through a 100μm strainer, then through a 40μm strainer, before pelleting by centrifugation for 10min at 1,000xg in a swinging bucket rotor. Cell pellets were separated by centrifugation for 1hr at 1,200xg on a discontinuous gradient of isotonic Percoll (75%, 50%, 0%) and FMCs were collected from the 75%/50% interphase. FMCs were washed in dPBS before being pelleted and resuspended in dPBS with 10% heat-inactivated fetal bovine serum (FBS). Yield was determined by hemocytometer and cells were immediately processed for antibody labeling and flow cytometry.

For ex vivo stimulation (Supplemental Fig.7), FMCs and astrocytes were separated from one hemisphere by centrifugation on a 4-step gradient of isotonic Percoll (75%/50%/30%/0%). FMCs were collected from the 75%/50% interphase, astrocytes were collected from the 50%/30% interphase, as described.[2,9] BVECs were isolated from the opposite hemisphere by dextran gradient centrifugation followed by Percoll gradient ultracentrifugation according to published protocols[2,9] and as detailed in the Reporting Summary.

Ovalbumin stimulation and qPCR

For ovalbumin stimulation, freshly isolated cells were plated in duplicate wells of v-bottom 96-well plates (10^5 cells/well) and adhered for 1hr in serum-free DMEM at 37C under 5%CO2. After adherence, media was aspirated and replaced with 1xdPBS containing 10%FBS with or without 100 micrograms/mL ovalbumin (Sigma-Aldrich). Ovalbumin stimulation was carried out for 20min at 37C before aspiration, washes, and overnight maintenance in DMEM+10%FBS at 37C under 5%CO2. The following day, viability was determined using a formazan cleavage assay (R&D Systems) and cells were snap frozen for RNA extraction and qPCR, as described.[2] In brief, RNA was extracted and purified from cells using the RNeasy blood and tissue kit (Qiagen). cDNA was synthesized from the purified RNA template using a commercially available kit (Applied Biosystems). cDNA (500ng) was amplified using Taqman Mastermix and Taqman probes on a StepOne plus instrument. Expression was determined by calculating ddCT, with the average dCT from LFD/SHAM cells as the reference group. For a list of Taqman probes used in these experiments, see Supplementary Table 1.

Immunofluorescence and imaging in decalcified skull preparations

For immunofluorescence detection of meningeal T cells, mice were transcardially perfused with PFA and denuded heads were postfixed for 24hr prior to mechanical thinning of the skull using a dremel tool and grinding wheel. Thinned-skull preparations were then decalcified in 0.3M EDTA for 6d at RT with shaking before dehydration in 30% sucrose in phosphate buffer. Dehydrated skulls were frozen in OCT in preparation for sectioning on the transverse plane at 20 micron thickness using a cryostat. Horizontal

sections were collected onto numbered slides (1 section per slide) and every other slide processed for detection of CD3e (Santa Cruz cat#sc20047; 1:1,000), laminin (Dako cat#Z009701, 1:500), and streptavidin-405 amplification of biotinylated anti-IL4 (clone BVD4-1D11, BD Bioscience; 1.0 microgram injected ICV for in vivo capture).

For analysis of the leptomeninges, CD3e+ T-cells were rarely observed along the superior sagittal sinus (SSS), as previously reported.[10] T-cells were therefore sampled from both limbs of the transverse sinus, starting from (but not including) the confluence of sinuses (COS), and ending above the bifurcation point for the petrosquamosal sinus and sigmoid sinus. For the choroid plexus, images were acquired from the dorsal third ventricle according to the atlas of Paxinos and Franklin.[7] In both regions, CD3e+ cells, IL4+ cells, and CD3e+/IL4+ double-positive cells were quantified along laminin-positive basement membranes. Although the image acquisition scheme was not designed for morphological analysis of laminin-labeled structures, the total area of laminin immunoreactivity did not differ between conditions. As a result, cell counts are reported without normalizing to laminin labeling area.

Immunofluorescence and 3D reconstruction of microglia

For immunofluorescence visualization of microglia, 40 micron coronal sections were cut throughout the rostrocaudal extent of the hippocampus as a 1:6 series using a freezing microtome (Leica), as described.[2] Free-floating sections were processed for detection of IBA1 (Wako, catalog #019-19741, 1:500) and CD68 (AbDSerotec, catalog #MCA1957, 1:500) or Arg1 (Santa Cruz catalog #sc271430, 1:500). Sections were washed after primary antibody incubation and incubated with fluorophore-conjugated

secondary antibodies (Invitrogen, Carlsbad, CA) before being mounted on Superfrost Plus slides. Slides were counterstained with DAPI before blinded imaging on a Zeiss 780 multiphoton microscope.

Cells were imaged in the dentate middle molecular layer above the enclosed blade of the dentate gyrus. Blood vessels were avoided and only cells with a ramified morphology were sampled for analysis. During image acquisition, IBA1+ cells with cut processes on the surface of the section were excluded, and the size of each stack in the z-axis was tailored for each cell to avoid truncating fine processes. Before analysis, each stack was double-checked for truncated processes by saturating the image and looking for abrupt terminations without the characteristic taper. Image stacks (5 cells/animal) were imported into Neurolucida 360 for semi-automated tracing and Sholl analysis. For IBA1/Arg1 double-labeling, (5) Arg1+ and (5) Arg1- cells were sampled from each animal. Total 3-dimensional length was averaged across cells to generate a single value for each animal. For Sholl analysis, the number of intersections at 1-micron intervals around the soma was averaged across cells sampled from a single animal and the the area under the curve was derived from the average Sholl profiles for statistical comparisons.

Supplemental References

1. McGee-Lawrence ME, Wenger KH, Misra S, Davis CL, Pollock NK, Elsalanty M, Ding K, Isales CM, Hamrick MW, Wosiski-Kuhn M, Arounleut P, Mattson MP, Cutler RG, Yu JC, Stranahan AM. Whole-Body Vibration Mimics the Metabolic Effects of Exercise in Male Leptin Receptor-Deficient Mice. Endocrinology. 2017 May 1;158(5):1160-1171.
2. Guo DH, Yamamoto M, Hernandez CM, Khodadadi H, Baban B, Stranahan AM. Visceral adipose NLRP3 impairs cognition in obesity via IL-1R1 on CX3CR1+ cells. J Clin Invest. 2020 Apr 1;130(4):1961-1976.

3. Erion JR, Wosiski-Kuhn M, Dey A, Hao S, Davis CL, Pollock NK, Stranahan AM. Obesity elicits interleukin 1-mediated deficits in hippocampal synaptic plasticity. J Neurosci. 2014 Feb 12;34(7):2618-31.
4. Mühlfeld C, Papadakis T, Krasteva G, Nyengaard JR, Hahn U, Kummer W. An unbiased stereological method for efficiently quantifying the innervation of the heart and other organs based on total length estimations. J Appl Physiol (1985). 2010 May;108(5):1402-9.
5. Nyengaard JR and Gundersen HJG. The isector: a simple and direct method for generating isotropic, uniform random sections from small specimens. Journal of Microscopy. 1992; 165:427-431.
6. Chi J, Wu Z, Choi CHJ, Nguyen L, Tegegne S, Ackerman SE, Crane A, Marchildon F, Tessier-Lavigne M, Cohen P. Three-Dimensional Adipose Tissue Imaging Reveals Regional Variation in Beige Fat Biogenesis and PRDM16-Dependent Sympathetic Neurite Density. Cell Metab. 2018 Jan 9;27(1):226-236.e3.
7. Paxinos G, Franklin KB, 2001. The mouse brain in stereotaxic coordinates, second Ed. San Diego, Academic Press.
8. Finkelman FD, Morris SC. Development of an assay to measure in vivo cytokine production in the mouse. Int Immunol. 1999 Nov;11(11):1811-8.
9. Yamamoto M, Guo DH, Hernandez CM, Stranahan AM. Endothelial Adora2a Activation Promotes Blood-Brain Barrier Breakdown and Cognitive Impairment in Mice with Diet-Induced Insulin Resistance. J Neurosci. 2019 May 22;39(21):4179-4192.
10. Ahn JH, Cho H, Kim JH, Kim SH, Ham JS, Park I, Suh SH, Hong SP, Song JH, Hong YK, Jeong Y, Park SH, Koh GY. Meningeal lymphatic vessels at the skull base drain cerebrospinal fluid. Nature. 2019 Aug;572(7767):62-66.

Supplementary Table 1. Taqman probes used for qPCR.

Gene	Taqman probe
Il1b	Mm00434228_m1
Il4	Mm00445259_m1
Il6	Mm00446190_m1
Ccl2	Mm00441242_m1
Tlr4	Mm00445273_m1
Tnf	Mm00443258_m1
Cidea	Mm00432554_m1
Prdm16	Mm00712556_m1
Ucp1	Mm01244861_m1
Ppargc1a	Mm01208835_m1
Pparg	Mm00440940_m1
Gapdh	Mm99999915_g1

Supplementary Table 2. Antibodies and dilutions used for immunohistochemistry and immunofluorescence.

Antibody	Dilution	supplier	cat #
UCP1	1:1,000	Abcam	ab10983
tyrosine hydroxylase	1:1,000	Millipore	AB152
F4/80	1:500	Santa Cruz	sc25830
IBA1	1:500	Wako Diagnostics	019-19741
CD68	1:500	Bio-Rad	MCA1957
Arg1	1:500	Santa Cruz	sc271430
CD3e	1:1,000	Santa Cruz	sc20047
laminin	1:500	Dako	Z009701

Supplementary Table 3. Antibody information for flow cytometry.

**Antigen**	**Dye**	**Clone**	**Supplier**	**cat #**
IL10Ra	DL350	1B1.3a	Novus Biologicals	NB100-63577
MHCII	BUV563	OX-6	BD Biosciences	749006
Ly6G	BV510	1A8	BioLegend	127633
CD169	BV605	3D6	BD Biosciences	566606
CD45	FITC	30-F11	BioLegend	103108
Arg1	PerCP	N/A	Novus Biologicals	NBP1-32731
TMEM119	PE	N/A	Abcam	ab225496
IL4Ra	PE-Cy7	I015F8	BioLegend	144805
CD11b	AF647	M1/70	BioLegend	101218
Ly6C	AF700	AL-21	BD Biosciences	562728
TLR4	APC-Cy7	HTA125	Novus Biologicals	NB100-56723
CD3e	PE	KT3.1.1	BioLegend	155608
CD4	BV785	RM4-5	BioLegend	100551
IL-4	AF488	11B11	BD Biosciences	557728
CD3e	Pacific Blue	KT3.1.1	BioLegend	155611
CD4	BV785	RM4-5	BioLegend	100551
CD45	FITC	30-F11	BioLegend	103108
CD11b	AF700	M1/70	BioLegend	101222
