# Supplementary material for: Beige adipocytes mediate the neuroprotective and anti-inflammatory effects of subcutaneous fat in obese mice
Source: Nat Commun. 2021 Jul 30;12:4623. doi: 10.1038/s41467-021-24540-8 (PMC8324783; doi:10.1038/s41467-021-24540-8)
Supplement: Supplementary file 2 — Reporting Summary [file 41467_2021_24540_MOESM2_ESM.pdf]

nature portfolio

Corresponding author(s): Stranahan AM

Last updated by author(s): Jun 17, 2021

Reporting Summary

Nature Portfolio wishes to improve the reproducibility of the work that we publish. This form provides structure for consistency and transparency in reporting. For further information on Nature Portfolio policies, see our Editorial Policies and the Editorial Policy Checklist.

Statistics

For all statistical analyses, confirm that the following items are present in the figure legend, table legend, main text, or Methods section.

n/a	Confirmed	
☐	☒	The exact sample size (*n*) for each experimental group/condition, given as a discrete number and unit of measurement
☐	☒	A statement on whether measurements were taken from distinct samples or whether the same sample was measured repeatedly
☐	☒	The statistical test(s) used AND whether they are one- or two-sided<br>*Only common tests should be described solely by name; describe more complex techniques in the Methods section.*
☒	☐	A description of all covariates tested
☐	☒	A description of any assumptions or corrections, such as tests of normality and adjustment for multiple comparisons
☐	☒	A full description of the statistical parameters including central tendency (e.g. means) or other basic estimates (e.g. regression coefficient) AND variation (e.g. standard deviation) or associated estimates of uncertainty (e.g. confidence intervals)
☐	☒	For null hypothesis testing, the test statistic (e.g. *F*, *t*, *r*) with confidence intervals, effect sizes, degrees of freedom and *P* value noted<br>*Give P values as exact values whenever suitable.*
☒	☐	For Bayesian analysis, information on the choice of priors and Markov chain Monte Carlo settings
☐	☒	For hierarchical and complex designs, identification of the appropriate level for tests and full reporting of outcomes
☒	☐	Estimates of effect sizes (e.g. Cohen's *d*, Pearson's *r*), indicating how they were calculated

Our web collection on statistics for biologists contains articles on many of the points above.

Software and code

Policy information about availability of computer code

Data collection	StereoInvestigator version 11.0 (Microbrightfield), Neurolucida 360 version 11.0 (Microbrightfield), FACSDiva version 8.0 (BD Bioscience), Guava InCyte version 2.6 (Millipore), Accuri C6 Plus software version 3.4 (BD Bioscience) Any-Maze version 6.34 (Stoelting), PClamp version 10 (Axon Instruments), StepOne Plus version 2.3 (Thermo Fisher)
Data analysis	Neurolucida Explorer version 11.0 (Microbrightfield), FlowJo version 10.6 (Treestar), Clampfit version 10 (Axon Instruments) GraphPad Prism version 8.0 (Prism); ImageJ version 1.53 (imagej.nih.gov)

For manuscripts utilizing custom algorithms or software that are central to the research but not yet described in published literature, software must be made available to editors and reviewers. We strongly encourage code deposition in a community repository (e.g. GitHub). See the Nature Portfolio guidelines for submitting code & software for further information.

Data

Policy information about availability of data

All manuscripts must include a data availability statement. This statement should provide the following information, where applicable:

- Accession codes, unique identifiers, or web links for publicly available datasets
- A description of any restrictions on data availability
- For clinical datasets or third party data, please ensure that the statement adheres to our policy

All source data shown in the main manuscript and Supplementary Figures will be published with the manuscript. Detailed step-by-step protocols are available on request from the corresponding author.

Field-specific reporting

Please select the one below that is the best fit for your research. If you are not sure, read the appropriate sections before making your selection.

☒ Life sciences ☐ Behavioural & social sciences ☐ Ecological, evolutionary & environmental sciences

For a reference copy of the document with all sections, see nature.com/documents/nr-reporting-summary-flat.pdf

Life sciences study design

All studies must disclose on these points even when the disclosure is negative.

Sample size	Sample sizes were determined using power analysis as follows: Step 1) Identify previous work comparing the same or related outcomes in Wt mice on LFD or HFD. Step 2) Calculate effect size (Cohen's d) for the effect of dietary obesity in Wt mice using the following formula: d= LFD mean - HFD mean / pooled standard deviation, where pooled standard deviation (stdev) = (LFDstdev+HFDstdev)/2. Step 3) Use Cohen's d to estimate sample sizes at beta=0.2 for the predetermined experimental design (1-way or 2-way ANOVA, as appropriate). Sample size estimations were calculated using WebPower (https://webpower.psychstat.org/wiki/).
Data exclusions	Histological criteria for transplant rejection were predetermined and followed previously published studies (Guo et al 2020). For all the transplant experiments in the manuscript, 9/107 transplant recipients exhibited transplant rejection (as shown in Supplementary Figure 4A-B) and these mice were excluded from the study. Data on the number of transplant rejections is reported in the Results section.
Replication	All experiments were carried out in balanced cohorts of (n=3-5) mice per condition. Group differences were consistent across cohorts indicating that the findings were reproducible. We internally replicated the effects of 4wk HFD on microglial activation in nontransgenic mice (Fig. 1E-G and Fig. 4B-E), and the effects of 4wk HFD on serum cytokines in nontransgenic mice (Fig.1D and Fig.5A). Although the data in Fig.4 and Fig.5 are from sham-operated mice (as opposed to the data from surgically naive animals in Fig.1), we considered the potential impact of sham surgery when presenting these results in the manuscript. Because serum cytokines and microglial activation were comparable between surgically naive animals and sham-operated animals, we consider this an internal replication.
Randomization	Mice were pseudorandomly assigned to generate body weight-balanced groups before the start of experimental diets (8wk old) and at the time of surgery (10wk or 18wk old). Within each diet or diet x surgery condition, mice were again pseudorandomized by body weight for experimental endpoints.
Blinding	Data acquisition was carried out blind to genotype and surgery, but could not be carried out blind to diet because of the visually evident dietary obesity phenotype. Data analysis was carried out blind to diet, genotype, and surgery.

Reporting for specific materials, systems and methods

We require information from authors about some types of materials, experimental systems and methods used in many studies. Here, indicate whether each material, system or method listed is relevant to your study. If you are not sure if a list item applies to your research, read the appropriate section before selecting a response.

Materials & experimental systems

n/a	Involved in the study	
☐	☒	Antibodies
☒	☐	Eukaryotic cell lines
☒	☐	Palaeontology and archaeology
☐	☒	Animals and other organisms
☒	☐	Human research participants
☒	☐	Clinical data
☒	☐	Dual use research of concern

Methods

n/a	Involved in the study	
☒	☐	ChIP-seq
☐	☒	Flow cytometry
☒	☐	MRI-based neuroimaging

Antibodies

Antibodies used	UCP1 (Abcam cat#ab10983), tyrosine hydroxylase (Millipore cat#AB152); F4/80 (Santa Cruz cat#sc25830); CD3e (Santa Cruz cat#sc20047); laminin (Dako cat#Z009701); IBA1 (Wako, cat#019-19741); CD68 (AbDSerotec, cat#MCA1957); Arg1 (Santa Cruz cat#sc271430)
Validation	For immunofluorescence and immunohistochemistry, in-house validation was performed by omission of 1ary antibodies for all antigens. External validation includes absence of labeling in models of microglia depletion (IBA1; Bruttger et al 2015 Immunity 43:92), absence of labeling in tissue specific Arg1 knockouts (Arg1, Stoermer et al 2012 J Immunol 189:4047); validated for immunofluorescence and flow by manufacturer (CD68), used by multiple groups with consistent anatomical staining pattern (see Stevens et al, 2007 Cell 131:1164, see also Schafer et al, 2012 Neuron 74:691; staining pattern consistent with pattern observed with same antibody by structured illumination microscopy, see Fumagalli et al, 2019 J Neuroinflammation 16:9); for laminin, same antibody used by multiple groups with consistent anatomical staining pattern (see Cha et al, 2014 Nature Communications 5:4952;

see also Bifari et al, 2015 Frontiers in Cellular Neuroscience 9:383); for CD3e, elimination of positive labeling after irradiation (CD3e, Titova et al 2014 Neuroscience 283:231); absence of binding on western blots from Ucp1-/- mice (Kazak et al, 2017 PNAS USA 114:7981); complete loss of TH expression following surgical or chemical denervation of adipose tissue (Fischer et al, 2019 Am J Physiol Endocrinol Metab 316:E487; Jiang et al, 2017 Cell Metab 26:686); and elimination of tissue F4/80+ macrophages following clodronate depletion (Chaves et al, 2013 J Immunol Res 836989).

Animals and other organisms

Policy information about studies involving animals; ARRIVE guidelines recommended for reporting animal research

Laboratory animals	Male mice were maintained on experimental diets beginning at 8wk old. For SAT transplantation experiments, male donor mice (6-8wk old) were maintained on standard chow (Teklad; see Supplementary Methods). Colony temperature was set at 22C and recorded temperatures ranged from 21-24C over the course of the experiments (humidity 40-60%). The colony room was maintained on a 12hr light:dark cycle (lights-on at 0600hr).
Wild animals	No wild animals were used in this study.
Field-collected samples	No field-collected samples were used in this study.
Ethics oversight	All experimental procedures followed NIH guidelines and were approved by the Institutional Animal Care and Use Committee at Augusta University.

Note that full information on the approval of the study protocol must also be provided in the manuscript.

Flow Cytometry

Plots

Confirm that:

- [x] The axis labels state the marker and fluorochrome used (e.g. CD4-FITC).
- [x] The axis scales are clearly visible. Include numbers along axes only for bottom left plot of group (a 'group' is an analysis of identical markers).
- [x] All plots are contour plots with outliers or pseudocolor plots.
- [x] A numerical value for number of cells or percentage (with statistics) is provided.

Methodology

Sample preparation	For isolation of forebrain mononuclear cells (FMCs; Fig.1e-g, Fig.4b-e, Fig.5i, and Fig.6b-c), brains were extracted from the skull after transcardial perfusion with saline. After removing the cerebellum and brainstem, forebrain hemispheres were bisected and midline white matter tracts were discarded. Each hemisphere was manually dissociated in an ice-cold Tenbroeck homogenizer containing 5.0mL sterile Dulbecco's phosphate-buffered saline (dPBS) supplemented with 0.2% D-glucose. Dissociated cells were passed through a 100 micron strainer, then through a 40 micron strainer, before pelleting by centrifugation for 10min at 1,000xg in a swinging bucket rotor. Cell pellets were separated by centrifugation for 1hr at 1,200xg on a discontinuous gradient of isotonic Percoll (75%, 50%, 0%) and FMCs were collected from the 75%/50% interphase. FMCs were washed in dPBS before being pelleted and resuspended in dPBS with 10% heat-inactivated fetal bovine serum (FBS). Yield was determined by hemocytometer and cells were immediately processed for antibody labeling and flow cytometry.<br>For ex vivo stimulation (Supplemental Fig.7), mice were terminally anesthetized with Isoflurane and sacrificed by decapitation. FMCs and astrocytes were separated from one hemisphere by centrifugation on a 4-step gradient of isotonic Percoll (75%/50%/30%/0%). FMCs were collected from the 75%/50% interphase and astrocytes were collected from the 50%/30% interphase. Brain vascular endothelial cells (BVECs) were isolated from the opposite hemisphere after manual dissocation in 5.0mL vascular isolation buffer (VIB; 15mM Hepes, 147mM NaCl, 4mM KCl, 3mM CaCl2, 12mM MgCl2). Cells were pelleted by centrifugation (5min at 1,000xg), resuspended in VIB, and layered on top of an equal volume of 15% dextran. Dextran gradients were separated by centrifugation at 1,500xg for 1hr at 4C in a swinging bucket rotor. After separation, the vascular pellet under the dextran layer was washed by resuspension, pelleted, and dissociated by agitation in VIB with 10% Accumax for 20min at RT. Dissociated cell suspensions were filtered through a 100m strainer, pelleted, and resuspended in 50% isotonic Percoll in preparation for ultracentrifugation (10min at 20,000xg with minimal acceleration and no brake). BVECs were collected from the upper phase, washed by resuspension, and pelleted in preparation for determination of yield by hemocytometer.<br>For collagenase digestion and isolation of stromal-vascular fraction (SVF) from adipose tissue (Fig.6c), transplanted SAT, resident SAT, and VAT were dissected, weighed, and washed in DMEM to remove hair and debris. Visible blood vessels were removed and discarded, then fat pads were minced and transferred to conical tubes. Dissociation buffer (1xdPBS with 2.0mg/mL collagenase IV and 20mg/mL BSA; 3x wt/vol) was added to each tube and samples dissociated for 30-40min at 37C on a shaker inside of a hybridization oven. After dissociation, 3x (vol/vol) DMEM+2%FBS was added to each tube. Cell suspensions were passed through a 100 micron strainer and pelleted by centrifugation at 250xg for 8min. The lipid layer and media were aspirated and discarded, and the SVF pellet was resuspended in 5mL DMEM+FBS. The SVF pellet was filtered through a 40 micron strainer, pelleted by centrifugation at 400xg for 10min, then washed 2x by repeated resuspension and centrifugation in preparation for flow cytometry.

Instrument	Flow cytometric data acqusition was carried out on a 5-laser BD LSRII in FACSDiva software version 8.1 (BD Bioscience), a Guava EasyCyte 5.0 with Incyte version 2.6, Millipore), or on an Accuri C6 flow cytometer with Accuri C6 Plus software (version 3.4, BD Bioscience).
Software	Data analysis was carried out in FlowJo version 10.6 (Treestar).
Cell population abundance	These data are shown as scatterplot graphs, with individual symbols representing cells from a single mouse. In some cases, these data are reported as mean+/-sem in the text of the Results section.
Gating strategy	Gating schematics are included in Figure 1E, Figure 5I, and Figure 6B.

☒ Tick this box to confirm that a figure exemplifying the gating strategy is provided in the Supplementary Information.
